# The phosphorylation status of eukaryotic elongation factor-2 indicates neural activity in the brain

**DOI:** 10.1186/s13041-021-00852-0

**Published:** 2021-09-15

**Authors:** Sang Ho Yoon, Woo Seok Song, Sung Pyo Oh, Young Sook Kim, Myoung-Hwan Kim

**Affiliations:** 1 Department of Physiology and Biomedical Sciences, Seoul National University College of Medicine, Seoul, 03080 Korea; 2 Neuroscience Research Institute, Seoul National University Medical Research Center, Seoul, 03080 Korea; 3 Seoul National University Bundang Hospital, Seongnam, 13620 Gyeonggi Korea

**Keywords:** Eukaryotic elongation factor-2, eEF2, Phosphorylation, Dephosphorylation, Neural activity, Brain

## Abstract

Assessment of neural activity in the specific brain area is critical for understanding the circuit mechanisms underlying altered brain function and behaviors. A number of immediate early genes (IEGs) that are rapidly transcribed in neuronal cells in response to synaptic activity have been used as markers for neuronal activity. However, protein detection of IEGs requires translation, and the amount of newly synthesized gene product is usually insufficient to detect using western blotting, limiting their utility in western blot analysis of brain tissues for comparison of basal activity between control and genetically modified animals. Here, we show that the phosphorylation status of eukaryotic elongation factor-2 (eEF2) rapidly changes in response to synaptic and neural activities. Intraperitoneal injections of the GABA A receptor (GABA_A_R) antagonist picrotoxin and the glycine receptor antagonist brucine rapidly dephosphorylated eEF2. Conversely, potentiation of GABA_A_Rs or inhibition of AMPA receptors (AMPARs) induced rapid phosphorylation of eEF2 in both the hippocampus and forebrain of mice. Chemogenetic suppression of hippocampal principal neuron activity promoted eEF2 phosphorylation. Novel context exploration and acute restraint stress rapidly modified the phosphorylation status of hippocampal eEF2. Furthermore, the hippocampal eEF2 phosphorylation levels under basal conditions were reduced in mice exhibiting epilepsy and abnormally enhanced excitability in CA3 pyramidal neurons. Collectively, the results indicated that eEF2 phosphorylation status is sensitive to neural activity and the ratio of phosphorylated eEF2 to total eEF2 could be a molecular signature for estimating neural activity in a specific brain area.

**Supplementary Information:**

The online version contains supplementary material available at 10.1186/s13041-021-00852-0.

## Main text

Understanding neural activity in specific brain areas is critical for unraveling brain circuitry controlling normal behavior and elucidating disease mechanisms underlying brain dysfunction. Neural activity markers provide opportunities to estimate circuit activity, and immediate early genes (IEGs), such as c-*fos*, *jun*, *arc*, and *zif268*, have been used as neural activity markers [1, 2]. However, detection of IEG protein expression requires translation of genes, which restricts temporal resolution of signals, typically taking tens of minutes to several hours [3]. In addition, due to the small amount of newly synthesized gene products, western blot analysis of altered IEG expression, especially reduction in the IEG protein levels, is challenging which limits the utility of IEGs in western blot analysis for estimation of basal neural activity.

We previously observed that administration of the GABA_A_R antagonist pentylenetetrazol or reduction of interneuron density resulted in dephosphorylation of eukaryotic elongation factor 2 (eEF2) in the mouse hippocampus [4]. eEF2 is a GTP-dependent translocase that promotes the ribosomal translocation of peptidyl-tRNA during polypeptide elongation [5]. eEF2 kinase (eEF2K) phosphorylates eEF2 at threonine 56, which suppresses eEF2 activity and results in inhibition of protein synthesis [6]. Reportedly, synaptic activity affects eEF2K activity in cortical neurons [7]. Furthermore, action potential-mediated and miniature synaptic activity-mediated neurotransmissions in cultured hippocampal neurons promote eEF2 dephosphorylation (activation) and eEF2 phosphorylation (inactivation), respectively [8]. However, the effects of neural activity on eEF2 phosphorylation in the brain tissue of living animals are unclear.

**Fig. 1:**
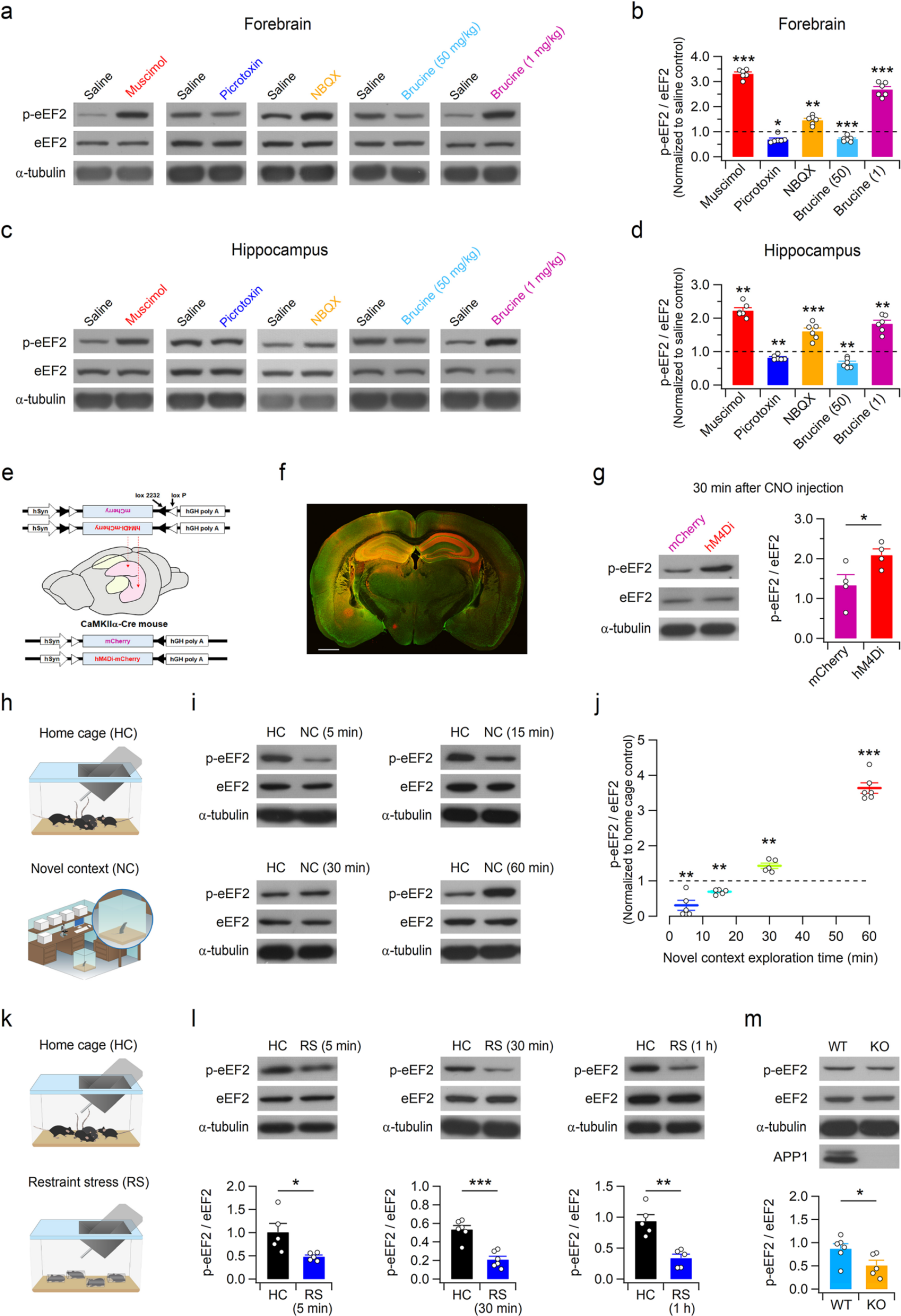
The eEF2 phosphorylation status is sensitive to synaptic and neuronal activities. **a**–**d** Administration of excitatory or inhibitory synaptic modulators rapidly modify the p-eEF2/eEF2 ratio in the forebrain and hippocampus. **a** Representative western blot images of p-eEF2 and eEF2 in the forebrain lysates obtained 1 h after drug or saline administration; α-tubulin was used as a loading control. **b** The ratios of phosphorylated eEF2 to total eEF2 (p-eEF2/eEF2) in the forebrain lysates obtained from drug-injected mice were normalized to saline-injected controls. Numbers in the parentheses indicate brucine doses (mg/kg) administered intraperitoneally. N = 6 mice for each group. Muscimol: t_(10)_ =  − 24.11, p = 3.42 × 10^−10^; NBQX: t_(10)_ =  − 3.65, p = 0.0045; Brucine (50): t_(10)_ = 5.10, p = 4.62 × 10^−4^; Brucine (1): t_(10)_ =  − 8.44, p = 7.30 × 10^−6^; Student’s *t*-test. Picrotoxin: U = 3.0, Z =  − 2.40, and p = 0.016 by Mann–Whitney test. **c** The p-eEF2 and total eEF2 protein levels in the hippocampal lysates obtained 1 h after drug administration. **d** Summary of the effects of synaptic modulators and blockers on the p-eEF2/eEF2 ratio in the mouse hippocampus. N = 6 mice for each group. Muscimol: U = 0.0, Z =  − 2.88, and p = 0.004; Picrotoxin: U = 1.0, Z =  − 2.72, and p = 0.006; Mann–Whitney test. NBQX: t_(10)_ =  − 5.89, p = 1.52 × 10^−4^; Brucine (50): t_(10)_ = 4.30, p = 0.0016; Brucine (1): t_(10)_ =  − 4.55, p = 0.0011; Student’s *t*-test. **e** Schematic diagram of Cre-dependent expression of mCherry or hM4Di-mCherry in the principal cells in the hippocampus. **f** Immunohistochemical staining of a coronal brain section showing mCherry expression in the hippocampal principal cells of CaMKIIα-Cre mice unilaterally injected with AAV2-hM4Di-mCherry. The section was co-immunostained with mCherry (red) and the neuronal marker NeuN (green). Scale bar, 1 mm. **g** Enhanced hippocampal p-eEF2 level (left) and the p-eEF2/eEF2 ratio (right) in the hM4Di-expressing mice compared to mCherry-expressing mice. N = 4 mice for each group. U = 1.0, Z =  − 2.02, and p = 0.043 by Mann–Whitney test. **h**-**j** Novel context exploration induces rapid dephosphorylation and subsequent phosphorylation of eEF2 in the hippocampus. Schematic diagram of the experimental design (**h**). **i** Representative western blot images of hippocampal p-eEF2 and eEF2 at various timepoints. **j** The hippocampal p-eEF2/eEF2 ratios in mice that explored a novel context were normalized to those of home cage controls and plotted against exploration time. N = 5–6 mice for each group. 5 min: t_(8)_ = 3.96, p = 0.0042; 15 min: t_(8)_ = 4.067, p = 0.0036; 30 min: t_(8)_ =  − 3.772, p = 0.0054; 60 min: t_(10)_ =  − 10.99, p = 6.66 × 10^−7^; Student’s *t*-test. **k**, **l** Restraint stress dephosphorylates eEF2 in the hippocampus. The experimental design (**k**), representative western blots (**l**, top), and quantification (**l**, bottom) showing reduced p-eEF2 levels in the hippocampus of mice exposed to restraint stress. N = 5–6 mice for each group. 5 min: t_(8)_ = 2.735, p = 0.0257; 30 min: t_(10)_ = 5.77, p = 1.79 × 10^−4^; 60 min: t_(8)_ = 4.840, p = 0.0013; Student’s *t*-test. **m** The basal levels of p-eEF2 proteins (top) and the p-eEF2/eEF2 ratio (bottom) are reduced in the hippocampus of Xpnpep1^−/−^ mice that lack aminopeptidase p1 (APP1) protein. N = 6 (Xpnpep1^+/+^) and 5 (Xpnpep1^−/−^) mice. t_(9)_ = 2.31 and p = 0.047 by Student’s *t*-test; U = 3.0, Z =  − 2.19, and p = 0.028 by Mann–Whitney test. **a**, **c**, **g**, **i**, **l**, and **m** The blots were cropped, and full blots are presented in the Additional file 1: Fig. S2

To investigate whether the eEF2 phosphorylation status is responsive to alteration in neural activity, the effects of GABA_A_R agonist muscimol and the antagonist picrotoxin on eEF2 phosphorylation were examined. Intraperitoneal (i.p.) injection of muscimol (1.5 mg/kg) and picrotoxin (2 mg/kg) produced opposite effects on eEF2 phosphorylation in both the hippocampus and forebrain without affecting total eEF2 levels (Fig. 1a–d and Additional file 1: Figs. S1 and S2); potentiation of GABAergic neurotransmission enhanced the phosphorylated eEF2 (p-eEF2) levels and suppression of inhibitory neurotransmission promoted eEF2 dephosphorylation. Consistent with this observation, the AMPAR blocker NBQX (10 mg/kg, i.p.) significantly enhanced eEF2 phosphorylation in both brain areas within 1 h after injection. These results indicate that altered neural activity bidirectionally modifies eEF2 phosphorylation status in the brain. We further examined eEF2 phosphorylation with the glycine receptor blocker brucine (Fig. 1a–d). Similar to picrotoxin, a high concentration (50 mg/kg) of brucine promoted eEF2 dephosphorylation in the mouse brain. Unexpectedly, a low concentration (1 mg/kg) of brucine significantly enhanced p-eEF2 levels, indicating this concentration of brucine decreases the activity of principal neurons in the hippocampus and forebrain presumably through the modulation of non-glycinergic neurotransmission (Additional file 1: Figs. S3 and S4).

As systemic administration of synaptic blockers affects neurons in the whole brain, their effects on the hippocampal eEF2 phosphorylation might originate from altered activity of excitatory and inhibitory neurons in other brain areas. To selectively manipulate hippocampal activity, inhibitory (hM4Di) designer receptors exclusively activated by designer drugs (DREADDs) were unilaterally expressed in the hippocampus of CaMKIIα-Cre mice (Fig. 1e). Strong mCherry signals were detected in the dendrites and somata of principal neurons in the ipsilateral hippocampus and their projections to contralateral hippocampus (Fig. 1f and Additional file 1: Fig. S5). We prepared ipsilateral hippocampal lysates from mCherry- and hM4Di-mCherry-expressing CaMKIIα-Cre mice 30 min after clozapine-N-oxide (CNO; 10 mg/kg, i.p.) injection. Compared to mCherry-expressing mice, p-eEF2 levels in the hippocampal lysates from hM4Di-expressing mice were significantly enhanced (Fig. 1g), indicating that suppression of hippocampal principal neuron activity is sufficient to promote eEF2 phosphorylation in the hippocampus.

We hypothesized that eEF2 phosphorylation status is regulated during physiological brain activity of living animals. Indeed, p-eEF2 level in the hippocampus was reduced and then significantly enhanced during the 1-h novel context exploration (Fig. 1h-j), indicating initial enhancement and subsequent reduction in the neural activity. As exploration of a novel environment induces both place cell firing and widespread synaptic depression in the rodent hippocampus [9, 10], gradual elevation of hippocampal p-eEF2 level might be associated with synaptic depression and resultant reduced neural activity. In contrast to novel context exploration, acute restraint stress significantly reduced hippocampal p-eEF2 at all (5, 30, and 60 min) timepoints (Fig. 1k, l), indicating an increase in synaptic strength induced by rapid action of stress hormones [11, 12]. We finally tested whether the eEF2 phosphorylation status is differently regulated by basal neural activity. Similar to RalBP1^−/−^ mice that exhibit reduced p-eEF2/eEF2 ratio [4], the hippocampal p-eEF2 levels were significantly reduced in Xpnpep1^−/−^ mice (Fig. 1m), which exhibit epileptic electroencephalogram rhythms and abnormally enhanced excitability of hippocampal CA3 pyramidal neurons [13, 14], indicating the eEF2 phosphorylation status is sensitive to basal neural activity.

Collectively, the present study shows that the phosphorylation status of eEF2 is sensitive to drug-induced synaptic modification, chemogenetic suppression of neuronal activity, environmental stimuli, and the resting activity of the specific brain area, and indicates that the p-eEF2/eEF2 ratio can be a molecular index to estimate neural activity in the brain. As multiple signaling mechanisms regulate eEF2K activity in neurons [7, 8], however, combined interaction of neural activity and signaling pathways on eEF2 phosphorylation needs further investigation.

## Supplementary Information

**Additional file 1.** Materials and methods, figures, and discussion.

## Data Availability

All raw data supporting the findings of this study are available upon reasonable request.

## Abbreviations

eEF2: Eukaryotic elongation factor-2
p-eEF2: Phosphorylated eukaryotic elongation factor-2
eEF2K: Eukaryotic elongation factor-2 kinase
IEG: Immediate early gene
DREADD: Designer receptors exclusively activated by designer drug
CNO: Clozapine-N-oxide
