# Supplementary material for: The phosphorylation status of eukaryotic elongation factor-2 indicates neural activity in the brain
Source: Mol Brain. 2021 Sep 15;14:142. doi: 10.1186/s13041-021-00852-0 (PMC8442277; doi:10.1186/s13041-021-00852-0)
Supplement: Supplementary file 1 — Additional file 1. Materials and methods, figures, and discussion. [file 13041_2021_852_MOESM1_ESM.pdf]

Additional file 1

The phosphorylation status of eukaryotic elongation factor-2 indicates neural activity in the brain

Sang Ho Yoon[1,2*], Woo Seok Song[1,2*], Sung Pyo Oh[1], Young Sook Kim[1], and Myoung-Hwan Kim[1,2,3#]

[1]Department of Physiology and Biomedical Sciences, Seoul National University College of Medicine, Seoul, 03080, Korea. [2]Neuroscience Research Institute, Seoul National University Medical Research Center, Seoul, 03080, Korea. [3]Seoul National University Bundang Hospital, Seongnam, Gyeonggi, 13620, Korea.

*These authors contributed equally to this work.
#E-mail: kmhwany@snu.ac.kr

Inventory of Additional file 1

Materials and methods

Figures (Fig. S1-S5)

Discussion

Materials and methods

Animals

All experiments were performed using male mice of C57BL/6N substrain purchased from Orient Bio (Seongnam, Gyeonggi, Korea), except for CaMKIIα-Cre (JAX stock #005359, The Jackson Laboratory, ME, USA) and Xpnpep1 mutant mice. CaMKIIα-Cre mice were transferred from Chung-Ang University (Seoul, Korea) and were backcrossed to C57BL/6N mice for at least 5 generations. Xpnpep1 mutant mice have been previously described [1]. The mice were backcrossed with two different inbred strains, C57BL/6J and 129S4/SvJae, for at least 10 generations before use. Xpnpep1$^{+/+}$ and Xpnpep1$^{-/-}$ mice were generated by intercrossing C57BL/6J and 129S4/SvJae heterozygous parents. Animals were group-housed (3−5/cage) in a specific pathogen-free facility and maintained in a climate-controlled room with free access to food and water under a 12-h light/12-h dark cycles with the light on at 7:00 AM. Animal maintenance and all animal experiments were performed under protocols approved by the Institutional Animal Care and Use Committee (IACUC) at Seoul National University.

Western blotting and immunohistochemistry

For western blot analysis, brains were removed and divided into left and right hemispheres. Hippocampi were removed from one of the two hemispheres and forebrains were collected from the other hemisphere by removing the cerebellum and brainstem. Hippocampi and forebrains were homogenized in a homogenization buffer (320 mM sucrose, 10 mM Tris-HCl, 5 mM EDTA, pH 7.4) containing phosphatase inhibitor cocktail (GenDEPOT, Katy, TX, USA, Cat. #P3200) and proteinase inhibitor cocktail (Sigma-Aldrich, St. Louis, MO, USA, Cat.

#P8340). The total protein concentrations in the homogenates was determined using the Bradford protein assay (Bio-Rad, Hercules, CA, USA). Proteins (10–12 μg) were separated using sodium dodecyl sulphate-polyacrylamide gel electrophoresis and transferred to nitrocellulose membranes. The membranes were blocked in Tris-buffered saline (TBST, 0.1% Tween 20) containing 5% skim milk for 30 min at room temperature, and then incubated with anti-eEF2 (Cat. #2332S, Cell Signaling Technology, Danvers, MA, USA), anti-p-eEF2 (Cat. #2331S, Cell Signaling Technology), or α-tubulin (Cat. #T5168, Sigma-Aldrich) antibodies for 1 h. After rinsing 3 times for 10 min with TBST, the membranes were incubated with horseradish peroxidase (HRP)-conjugated secondary antibodies for 1 h at room temperature and rinsed 3 times for 10 min with TBST. Antibody binding was visualized using enhanced chemiluminescence (GE Healthcare, Chalfont St Giles, UK). Western blot analyses were repeated at least 3 times, and band intensities were quantified using MetaMorph software (Molecular Devices, San Jose, CA, USA).

Immunohistochemistry and confocal microscopy were conducted as previously described [2]. Briefly, mice were deeply anesthetized with a mixture of Zoletil (50 mg/kg, i.p) and Xylazine (1 mg/kg, i.p), and underwent transcardial perfusion with heparinized (10 U/mL) phosphate-buffered saline (PBS) and a fixative containing 4% (w/v) paraformaldehyde (PFA) in PBS. Brains were removed from the skull, post-fixed (4% PFA) for 12 h at 4°C and sliced into 60-μm-thick sections using a vibratome (VT1200S, Leica, Wetzlar, Germany). The sections were permeabilized with 0.3% (v/v) Triton X-100 in PBS, incubated in a blocking buffer (5% normal goat serum, 5% horse serum, 5% donkey serum, 0.5% BSA in PBS) for 2 h, and incubated with primary antibodies (anti-mCherry, Abcam, Cat. #ab167453; anti-NeuN, Millipore, Cat. #MAB377) overnight at 4 °C. Next, sections were incubated with fluorescence (FITC or Cy3, Jackson ImmunoResearch Laboratories, West Grove, PA, USA) conjugated

secondary antibodies for 3 h at room temperature. Between each step, the sections were rinsed 3 times for 10 min with PBS. Sections were mounted on glass slides with coverslips and images were acquired using an TCS SP8 confocal microscope and Leica Application Suite X (Leica, Wetzlar, Germany).

Surgery and stereotaxic injection

Mice (6−7 weeks of age) were deeply anesthetized with a mixture of Zoletil (50 mg/kg, i.p) and Xylazine (1 mg/kg, i.p) and were placed in a stereotaxic device (Stoelting, Wood Dale, IL, USA). The skin was cut over the midline and craniotomies were performed on two sites unilaterally over the hippocampus. Purified AAV2/hSyn-DIO-mCherry (titration 4.9×10^{12} genome copies/mL; #50459, Addgene, Watertown, MA, USA) or AAV2/hSyn-DIO-hM4Di-mCherry (titration 5.3×10^{12} genome copies/mL; #44362, Addgene) were serially injected (1 μL/site) into the dorsal (−1.94 anteroposterior, −1.5 mediolateral, +1.9 dorsoventral from the Bregma) and ventral hippocampi (−2.92 anteroposterior, −2.8 mediolateral, +3.5 dorsoventral from the Bregma) in the same hemisphere at a rate of 100 nL/min using a Hamilton syringe. The needle (33 gauge) was left in place for 10 min after each injection to allow the diffusion of the viral solution, and then slowly retracted from the brain. Experiments were performed 3 weeks post injection to allow sufficient time for recovery and viral expression.

Drugs

Muscimol and brucine were purchased from Sigma-Aldrich. Picrotoxin, and CNO were purchased from Tocris (Bristol, UK).

Novel context exploration and restraint stress

Novel context exploration was conducted in the behavior testing room equipped with work benches, shelves, and cabinets. Mice were transferred from their home cages in the vivarium and individually placed in a square-type acrylic chamber (40 × 40 × 40 cm) containing clean bedding. Mice were allowed to explore the novel context for different amounts (5, 15, 30, and 60 min) of time. Restraint stress was conducted with well-ventilated 50-mL syringes. Mice were placed in the restraint tubes for 5, 30, or 60 min in their home cages. During the restraint, mice were able to move their head and anterior limbs but not the body and hindlimbs. Immediately after novel context exploration or restraint stress, brains were removed, snap-frozen, and stored at −80 °C until use.

Electrophysiology

Electrophysiological recordings in the hippocampal slices were performed according to the protocol described previously [2]. Briefly, hippocampal slices (400 μm-thick) were prepared from 4- to 5-week-old male mice using a vibratome. (VT1000S; Leica, Germany) in ice cold dissection buffer (in mM: sucrose 230, $NaHCO_3$ 25, KCl 2.5, NaH_2PO_4 1.25, D-glucose 10, Na-pyruvate 2, $MgCl_2$ 3.5, and $CaCl_2$ 0.5 bubbled with 95% O_2/5% CO_2), and then recovered for 1 h at 36°C in the artificial cerebrospinal fluid (aCSF; in mM: NaCl 125, $NaHCO_3$ 25, KCl 2.5, NaH_2PO_4 1.25, D-glucose 10, $MgCl_2$ 1.3, and $CaCl_2$ 2.5).

During the recording, slices were continuously perfused with the heated (30°C) aCSF. Signals were low-pass filtered at 2.8 kHz and digitized at 10 kHz using a MultiClamp 700B amplifier (Molecular Devices, USA) and a Digidata 1440A digitizer (Molecular Devices, USA).

Whole-cell patch clamp currents of CA1 neuron were recorded at the holding potential of 0

mV using a pipette (3–4 MΩ) solution containing (in mM) Cs-gluconate 100, TEA-Cl 10, CsCl 10, NaCl 8, HEPES 10, Mg-ATP 4, Na-GTP 0.3, and EGTA 10 (with pH 7.25, 290 mOsm). The $GABA_AR$ blocker bicuculline (10 μM) and the NMDAR blocker AP-5 (50 μM) were included in the aCSF. Recordings were started 10 min after establishment of the whole-cell configuration. The series resistance (< 10 MΩ) and seal resistance (> 1 GΩ) were monitored before and after recordings by applying a short (50 ms) hyperpolarization voltage pulse (−5 mV), and the data were discarded if the resistance changed by more than 20% during the recording.

Field excitatory postsynaptic potentials (fEPSPs) were recorded in a submerged chamber. The recording pipette (3–4 MΩ) was filled with aCSF and was placed in the CA1 stratum radiatum. Schaffer-collateral axons were stimulated every 20 s using a broken pipette (~ 0.5 MΩ) containing aCSF. The stimulation intensity was adjusted to yield 40% of the maximum response, and recordings were started 10 min after establishment of stable baseline response.

All data were analyzed using pClamp 10.2 software (Molecular Devices, USA) and custom macros written in Igor Pro 6 (WaveMetrics).

Statistical analysis

Statistical analyses were performed using Igor Pro (WaveMetrics) and SPSS (IBM, Armonk, NY, USA). The normality of the collected data was determined using the Shapiro-Wilk test. Unpaired, two-tailed Student's *t*-test and Mann-Whitney test were used to compare normally and non-normally distributed samples, respectively. None of the animals were excluded from analysis. All bar graphs in the figures show the mean ± standard error of the mean (SEM).

Figures

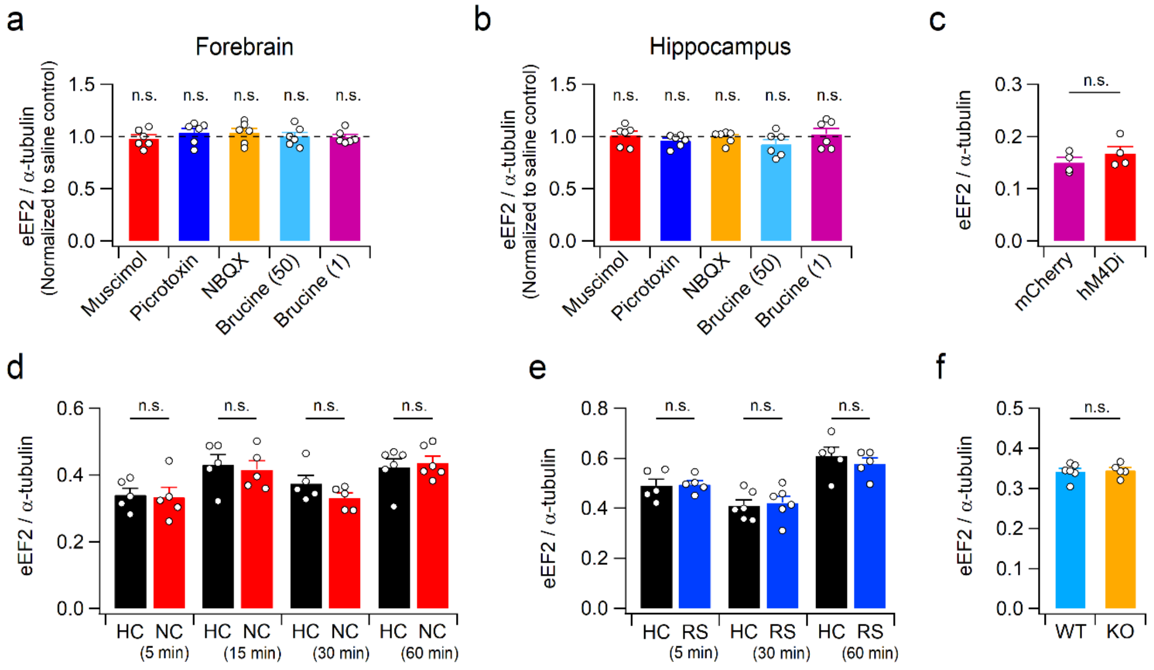

Fig. S1: Quantification of total eEF2 levels in forebrain and hippocampal lysates. (a, b) Total eEF2 level in the forebrain (a) and hippocampus (b) were not changed by excitatory or inhibitory synaptic modulators. The protein level of eEF2 was normalized to that of α-tubulin, and the relative expression level of eEF2 in drug-injected mice compared to saline-injected controls is summarized. Numbers in the parentheses indicate brucine doses (mg/kg) administered intraperitoneally. N = 6 mice for each group. (a) Muscimol: $t_{(10)} = 0.283$, $p = 0.783$; NBQX: $t_{(10)} = -0.559$, $p = 0.589$; Picrotoxin: $t_{(10)} = -0.583$, $p = 0.573$; Brucine (50): $t_{(10)} = -0.020$, $p = 0.985$; Brucine (1): $t_{(10)} = 0.055$, $p = 0.957$; Student's *t*-test. (b) Muscimol: $t_{(10)} = -0.172$, $p = 0.867$; NBQX: $t_{(10)} = 1.223$, $p = 0.249$; Picrotoxin: $t_{(10)} = 0.164$, $p = 0.873$; Brucine (50): $t_{(10)} = 1.200$, $p = 0.258$; Brucine (1): $t_{(10)} = -0.273$, $p = 0.790$; Student's *t*-test. (c) Bar graphs represent the total eEF2 level in hippocampal lysates obtained 30 min after CNO injection from mCherry- and hM4Di-expressing mice. U = 5.0, Z = –0.866, and p = 0.386 by Mann-Whitney test. (d) The total eEF2 levels in the hippocampus was not affected by novel context exploration. N = 5-6 mice for each group. 5 min: $t_{(8)} = 0.195$, $p = 0.859$; 15 min: $t_{(8)} = 0.372$, $p = 0.719$; 30 min: $t_{(8)} = 1.520$, $p = 0.167$; 60 min: $t_{(10)} = -0.413$, $p = 0.688$; Student's *t*-test. (e) Normal eEF2 level in the hippocampus of mice exposed to acute restraint stress. N = 5-6 mice for each group. 5 min: $t_{(8)} = -0.178$, $p = 0.863$; 30 min: $t_{(10)} = -0.323$, $p = 0.753$; 60 min: $t_{(8)} = 0.718$, $p = 0.492$; Student's *t*-test. (d, e) HC, home cage; NC, novel context; RS, restraint stress. (f) The hippocampal eEF2 level in Xpnpep1 WT and KO mice was quantified and summarized. $t_{(9)} = -0.218$ and $p = 0.831$ by Student's *t*-test.

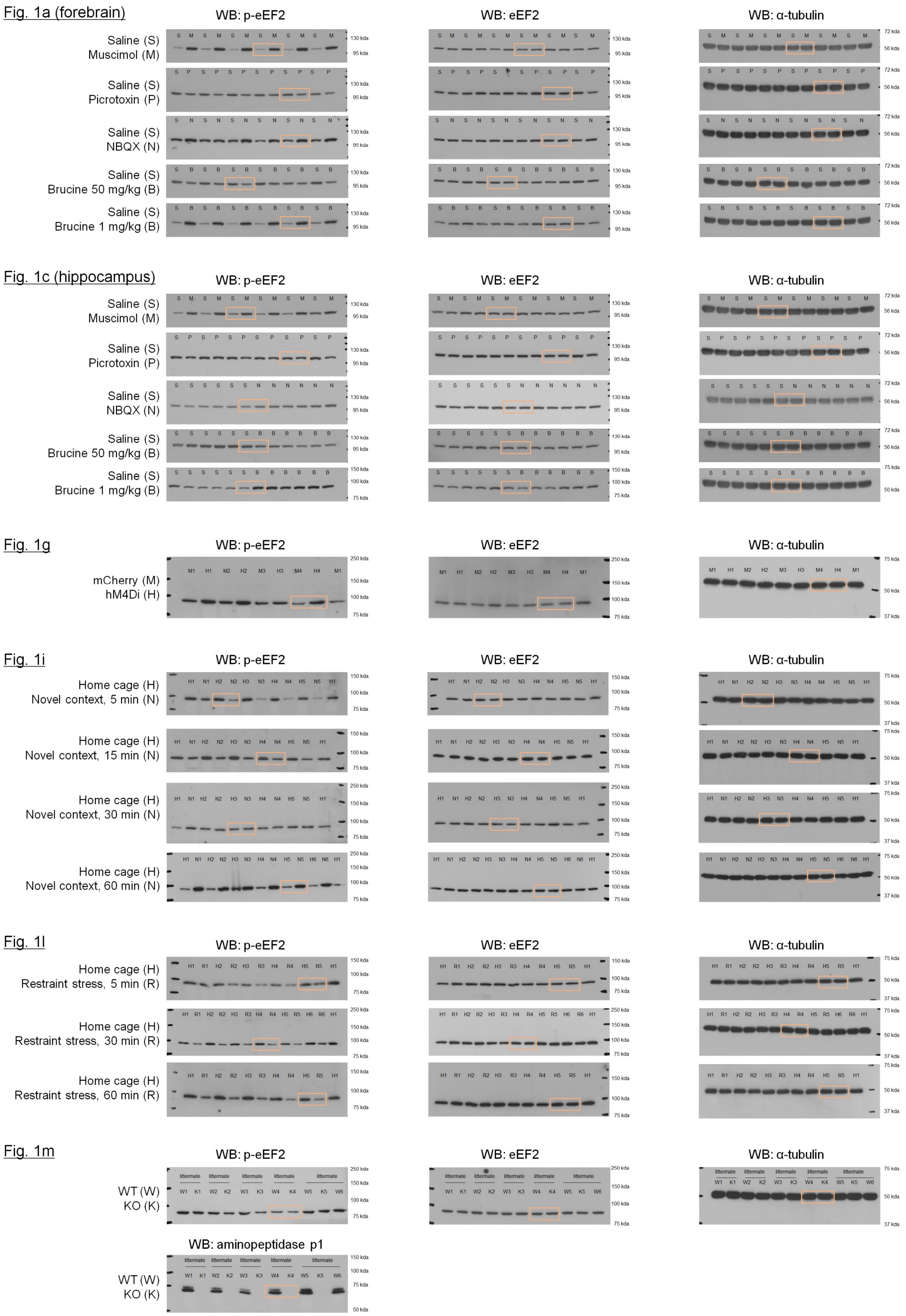

Fig. S2: Uncropped western blot images presented in the main figure.

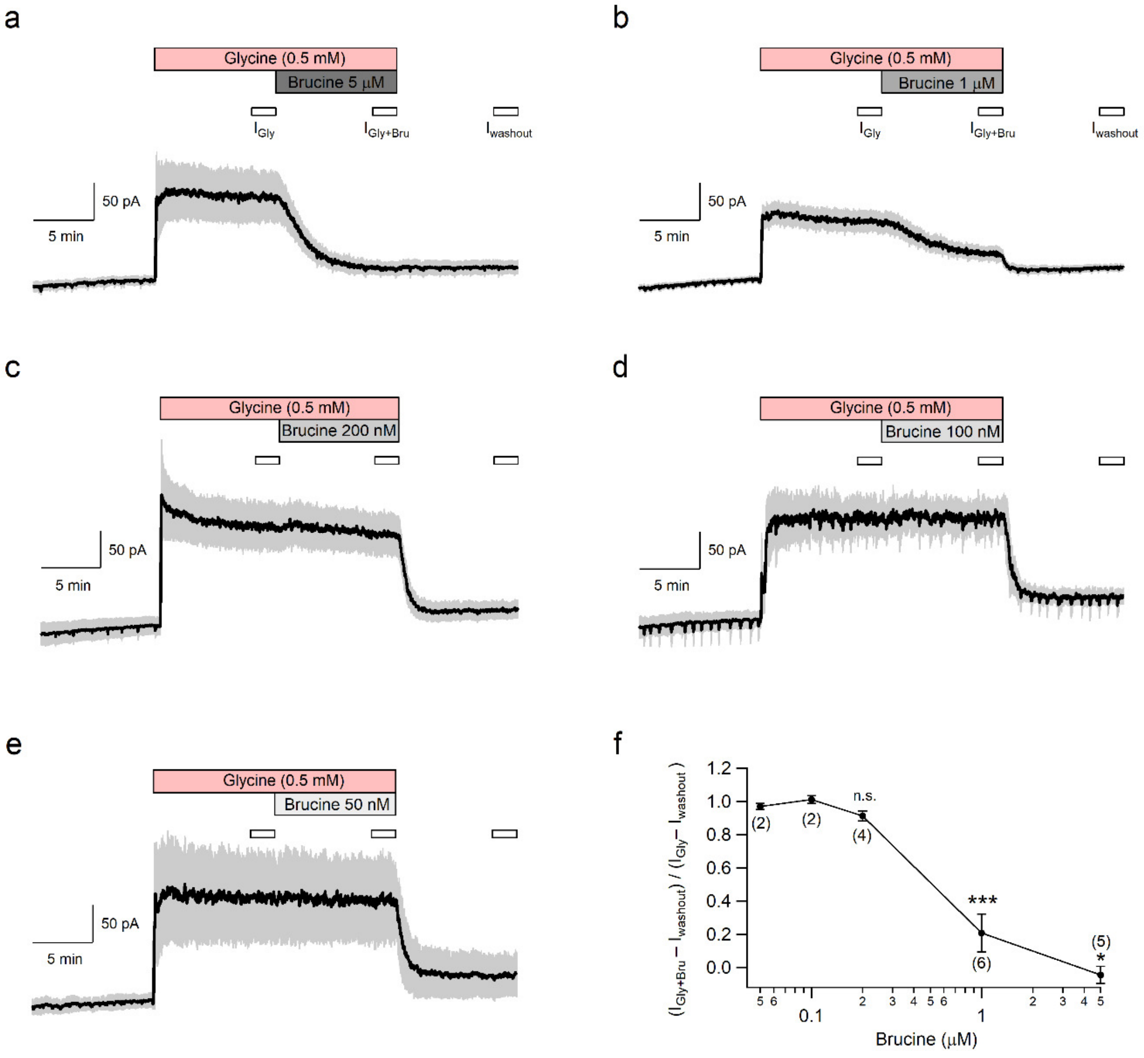

Fig. S3: Dose-dependent inhibition of the glycine-induced current by brucine. (a-e) Average time course of membrane currents showing the effects of various concentrations of brucine on glycine (500 µM)-induced outward current recorded in CA1 neurons at the holding potential of 0 mV in the presence of bicuculline (10 µM) and AP-5 (50 µM) in the aCSF. Brucine was applied during the last 10 min (indicated by horizontal bars) of the glycine perfusion (20 min). Black lines represent average traces, gray lines indicate standard error of the mean (SEM). (f) Dose-response relationship of the inhibitory effect of brucine on glycine-induced currents. Numbers in parentheses indicate number of data points. n.s., not significant, $p = 0.085$, $t_{(3)} = 2.53$; ***$p = 0.000994$, $t_{(6)} = 6.88$; *$p = 0.0164$, $t_{(4)} = 3.979$; Paired *t*-test [$(I_{Gly} - I_{washout})$ vs $(I_{Gly+Bru} - I_{washout})$].

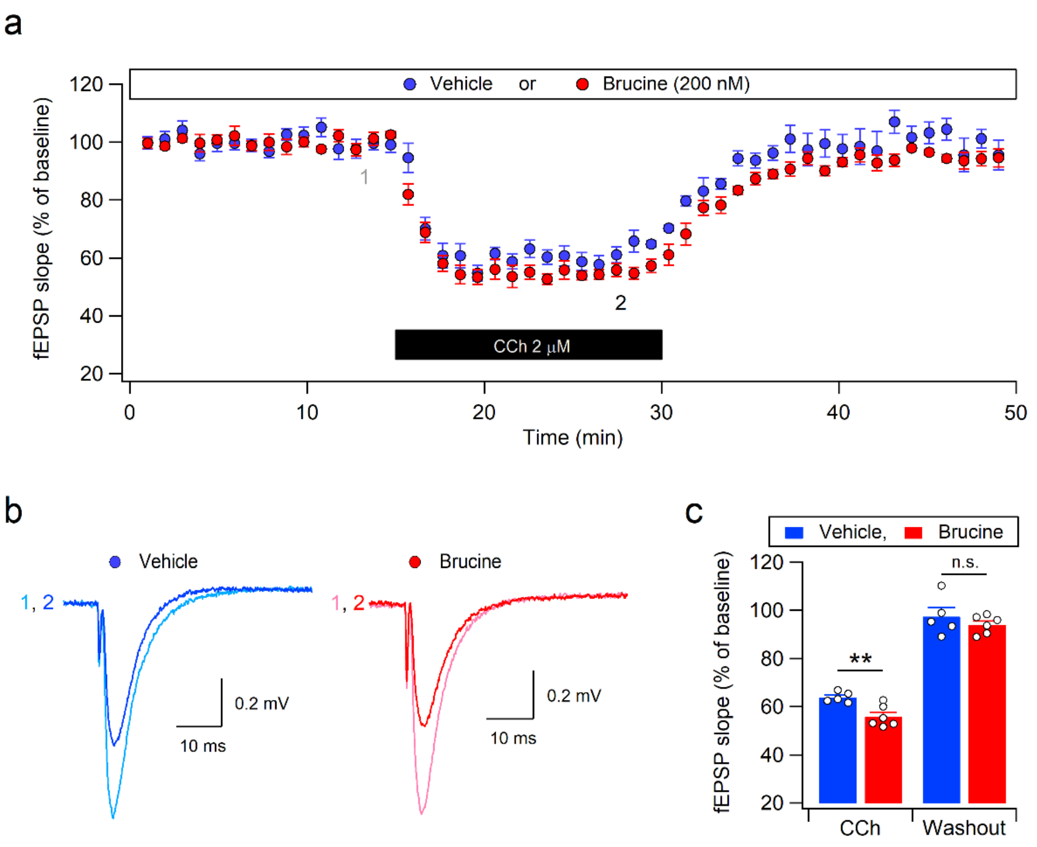

Fig. S4: A low concentration of brucine facilitates carbachol (CCh)-induced depression of excitatory synaptic transmission. (a) The initial slopes of fEPSPs measured at the Schaffer collateral-CA1 synapses in the absence or presence of brucine are plotted against time as a percentage of baseline. After 15 min of baseline recording, slices were perfused with CCh (2 μM) for 15 min. (b) Representative traces of fEPSPs obtained at baseline (1) and CCh perfusion (2). (c) The normalized slopes of fEPSP measured during CCh perfusion (25–30 min) and washout (45–50 min) are summarized. N = 5 (vehicle) and 6 (brucine) slices. CCh: $t_{(9)} = 3.635$, $p = 0.0054$; washout: $t_{(9)} = 0.904$, $p = 0.389$; Student's *t*-test.

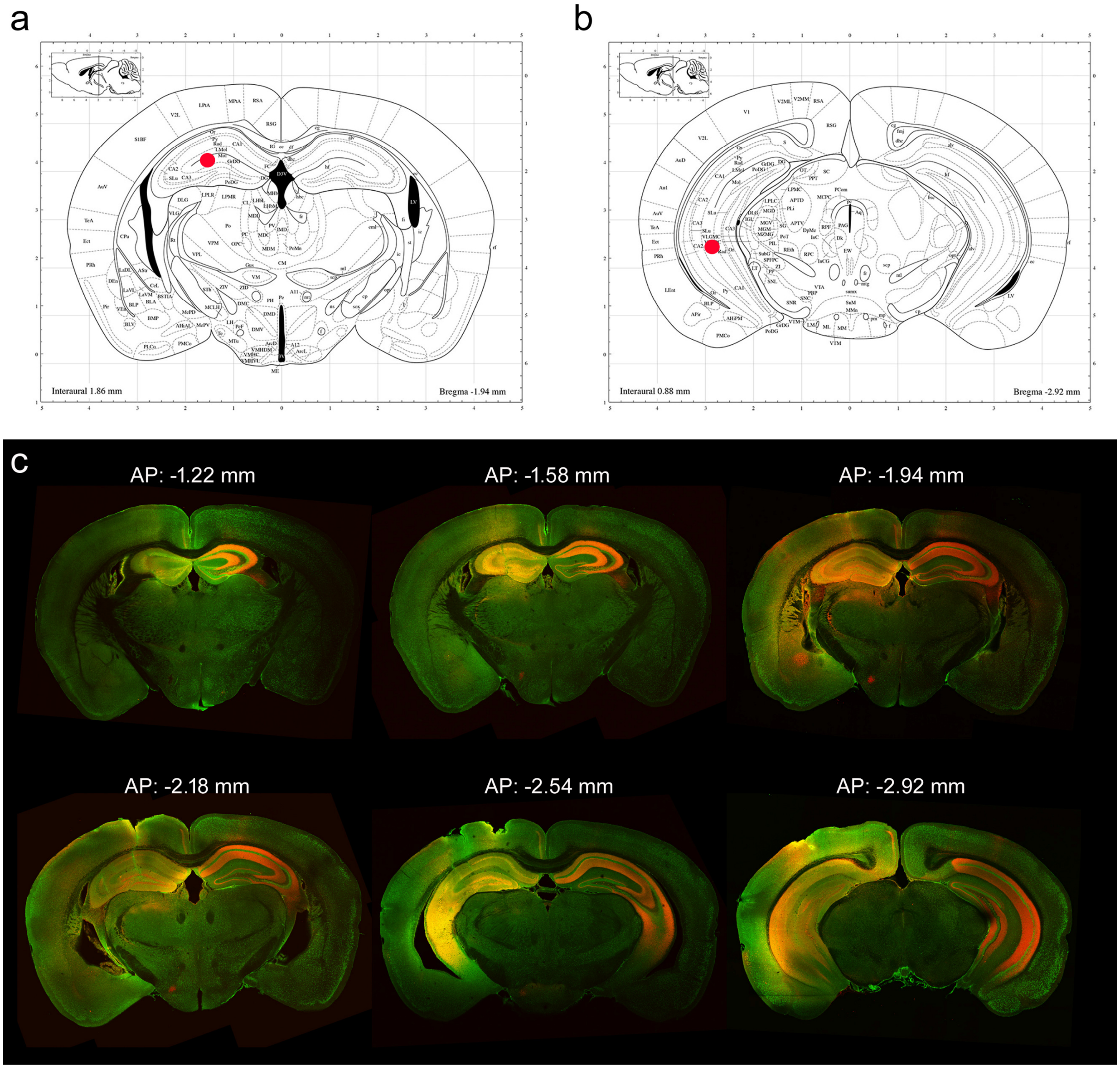

Fig. S5: The distribution of hM4Di-mCherry in the CaMKIIα-Cre mouse. (a, b) Microinjections of adeno-associated viruses were directed to the dorsal (a) and ventral (b) regions (red circles) of the hippocampus unilaterally. (c) Series of mCherry immunofluorescent images of coronal brain sections show the distribution of cells expressing mCherry (red) in the CaMKIIα-Cre mouse unilaterally injected with AAV2-hM4Di-mCherry. mCherry signals are detected in somata of principal cells in the ipsilateral hippocampus and axonal projections to the contralateral hippocampus. The neuronal marker NeuN (green) was used for the identification of brain regions. Numbers indicate anterior/posterior (AP) coordinates to Bregma.

Discussion

The present study demonstrates that eEF2 phosphorylation status, which is easily detectable using western blotting, is sensitive to drug-induced enhancement and suppression of neural activities. In addition, dephosphorylation of eEF2 was detected in the hippocampus of mice exposed to novel context or restraint stress within 5 min. Collectively, these results indicate that the effects of synaptic and neural activities on the eEF2 phosphorylation status in vivo are rapid and bidirectional. Because eEF2K regulation of eEF2 does not require transcription or translation, and the activity of eEF2 controls translation of proteins including Arc [3], the eEF2 phosphorylation status appears to indicate neural activity faster than protein expression of immediate early genes (IEGs) in the brain [4-7]. However, transcription of some IEGs is rapid such that intranuclear *arc* and *zif268* mRNAs were detected in the rat hippocampus by fluorescent in-situ hybridization (FISH) within 2 min after electroconvulsive shock [8]. These IEG mRNAs were continued to increase by 5 min, and they were subsequently disappeared from the nucleus and accumulated in the cytoplasm by 30 min. In addition, intranuclear *arc* mRNA was detected by FISH at 5 min after novel context exploration [8]. Similarly, elevation of c-*fos* mRNA was detected by northern blotting at 15 min and reached a maximum at 60 min after intraperitoneal injection of the convulsant metrazole [5]. Considering the time required for the flux of intraperitoneally administered drugs into the brain, transcription of c-*fos* might be initiated earlier than 15 min. Thus, the mRNA rather than protein level of IEGs seems to indicate neural activity with higher temporal resolution.

Intriguingly, the phosphorylated eEF2 (p-eEF2) level in the mouse hippocampus rapidly decreased and subsequently increased during novel context exploration (Fig. 1i, j). These results suggest that overall neural activity in the hippocampus is enhanced rapidly by exposure to a novel context and subsequently decreases below the baseline level as the animals habituate

to the environment. This finding appears contradictory to the previous observation of a strong induction of *arc* mRNA in the hippocampus of rats that explored a novel context for 1h [9]. As newly synthesized intranuclear *arc* mRNAs are transported to the cytoplasm and then to the dendritic processes [6, 8], cytoplasmic and dendritic *arc* mRNA signals in the CA1 region of the animal might not indicate enhanced neuronal activity immediately after or during the whole period of novel context exploration. Therefore, enhanced IEG expression does not necessarily represent enhanced neural activity at a specific timepoint. Instead, histochemical detection of IEG transcripts or proteins enables identification of activated neurons and an estimation of the activity history of labelled neurons [8]. Meanwhile, the phosphorylation status of eEF2 might indicate the activity of a neuronal population at a given timepoint but not the trajectory of overall neural activity. Consistent with intranuclear *arc* mRNA signals and enhanced activity of hippocampal place cells [8, 10, 11], the hippocampal p-eEF2 level immediately after novel context exploration for 5 and 15 min was significantly decreased. Considering that exposure of mice to a novel environment induces widespread and pathway-specific de novo synaptic depression in the hippocampus [12], spatial representations of novel context and synaptic depression exert opposite effects on neural activity in the hippocampus. Yet, the mechanism underlying enhanced p-eEF2 level in mice exposed to a novel context for 30 and 60 min remains unknown.

In contrast to novel context exploration, restraint stress for different amounts of time (5, 30, and 60 min) produced uniform changes in the pattern of eEF2 phosphorylation in the mouse hippocampus. This decrease in hippocampal p-eEF2 level implies enhanced neural activity during restraint stress. Although the brain mechanisms that translate stressful stimuli into activation of the hypothalamic-pituitary-adrenal (HPA) axis are incompletely understood, glucocorticoids released from the adrenal cortex during stress alter neural activity in various

brain regions, including the hippocampus, through binding to the mineralocorticoid receptors and glucocorticoid receptors [13]. Prolonged or severe stress suppresses long-term potentiation (LTP) and facilitates long-term depression (LTD) in the hippocampus [14, 15], while acute and mild stress can enhance neuronal excitability and excitatory synaptic transmission [13, 16]. Reportedly, acute immobilization or restraint stress for 45 and 30 min enhances the phosphorylation of calcium/calmodulin-dependent protein kinase II (CaMKII) and surface expression of Ca^{2+}-permeable AMPA receptors (AMPARs), respectively, in the hippocampus [17, 18]. Interestingly, the acute stress-induced up-regulation of phospho-CaMKII was blocked by pre-treatment with the AMPAR blocker NBQX in rats [17]. Collectively, these results support our observation that enhanced neural activity in response to acute stress results in dephosphorylation of eEF2 in the hippocampus. Similar to our findings, a recent study showed that acute restraint stress induces phosphorylation of the extracellular signal-regulated kinase (ERK) in the prefrontal cortex (PFC) of mice [19]. As glutamate-mediated activation of NMDA receptors (NMDARs) induces phosphorylation of ERK [20, 21], the enhanced level of phosphorylated ERK (p-ERK) is considered to indicate activation of neurons in the brain [22]. Compared to eEF2, phosphorylation of ERK seems to require stronger activation of neurons for detection by western blotting, as enhanced p-ERK level in the PFC were detected by 30-min restraint but not by 10-min restraint stress [19]. Similarly, a subconvulsant dose of pentylenetetrazol selectively dephosphorylated eEF2 without affecting p-ERK level in the mouse hippocampus [23]. In addition, we did not observe detectable changes in hippocampal p-ERK level in RalBP1 mutant mice and Xpnpep1 KO mice [1, 23], while p-eEF2 level was significantly reduced in both mutant mice. RalBP1 mutant mice exhibit reduced synaptic inhibition in CA1 neurons [23], and Xpnpep1 mutant mice exhibit epileptic electroencephalogram rhythms and enhanced neuronal excitability in CA3 neurons [1, 2]. These observations indicate that the phosphorylation status of eEF2 is more sensitive than that

of ERK in the brain of living animals.

Our study further predicts a reduction of neural activity by a low concentration (1 mg/kg, i.p.) of brucine. Reportedly, the binding affinity (IC50) of brucine for the glycine receptor measured by a binding assay is 0.072 μM [24]. In addition, brucine is an allosteric ligand for muscarinic acetylcholine receptors (mAChRs) and enhances the affinities for acetylcholine and carbachol (CCh) on mAChRs [25]. In our slice electrophysiology, concentrations of brucine lower than 200 nM had no effect on glycine-induced currents in hippocampal CA1 neurons, while 200 nM brucine facilitated CCh-induced depression of excitatory synaptic transmission. Although the brain concentrations of brucine achieved by intraperitoneal injections of 1 and 50 mg/kg brucine are unknown, our study provides experimental evidence that the phosphorylation status of eEF2 can be a useful marker to estimate neural activity in vivo.

References

1. Bae YS, Yoon SH, Han JY, Woo J, Cho YS, Kwon SK, Bae YC, Kim D, Kim E, Kim MH: **Deficiency of aminopeptidase P1 causes behavioral hyperactivity, cognitive deficits, and hippocampal neurodegeneration.** *Genes Brain Behav* 2018, **17:**126-138.
2. Yoon SH, Bae YS, Oh SP, Song WS, Chang H, Kim MH: **Altered hippocampal gene expression, glial cell population, and neuronal excitability in aminopeptidase P1 deficiency.** *Sci Rep* 2021, **11:**932.
3. Park S, Park JM, Kim S, Kim JA, Shepherd JD, Smith-Hicks CL, Chowdhury S, Kaufmann W, Kuhl D, Ryazanov AG, et al: **Elongation factor 2 and fragile X mental retardation protein control the dynamic translation of Arc/Arg3.1 essential for mGluR-LTD.** *Neuron* 2008, **59:**70-83.
4. Yamagata K, Andreasson KI, Kaufmann WE, Barnes CA, Worley PF: **Expression of a mitogen-inducible cyclooxygenase in brain neurons: regulation by synaptic activity and glucocorticoids.** *Neuron* 1993, **11:**371-386.
5. Morgan JI, Cohen DR, Hempstead JL, Curran T: **Mapping patterns of c-fos expression in the central nervous system after seizure.** *Science* 1987, **237:**192-197.
6. Lyford GL, Yamagata K, Kaufmann WE, Barnes CA, Sanders LK, Copeland NG, Gilbert DJ, Jenkins NA, Lanahan AA, Worley PF: **Arc, a growth factor and activity-regulated gene, encodes a novel cytoskeleton-associated protein that is enriched in neuronal dendrites.** *Neuron* 1995, **14:**433-445.
7. Sagar SM, Sharp FR, Curran T: **Expression of c-fos protein in brain: metabolic mapping at the cellular level.** *Science* 1988, **240:**1328-1331.
8. Guzowski JF, McNaughton BL, Barnes CA, Worley PF: **Environment-specific expression of the immediate-early gene Arc in hippocampal neuronal ensembles.** *Nat Neurosci* 1999, **2:**1120-1124.

9. Temple MD, Worley PF, Steward O: **Visualizing changes in circuit activity resulting from denervation and reinnervation using immediate early gene expression.** *J Neurosci* 2003, **23:**2779-2788.

10. Wilson MA, McNaughton BL: **Dynamics of the hippocampal ensemble code for space.** *Science* 1993, **261:**1055-1058.

11. Frank LM, Stanley GB, Brown EN: **Hippocampal plasticity across multiple days of exposure to novel environments.** *J Neurosci* 2004, **24:**7681-7689.

12. Ashby DM, Floresco SB, Phillips AG, McGirr A, Seamans JK, Wang YT: **LTD is involved in the formation and maintenance of rat hippocampal CA1 place-cell fields.** *Nat Commun* 2021, **12:**100.

13. Krugers HJ, Hoogenraad CC, Groc L: **Stress hormones and AMPA receptor trafficking in synaptic plasticity and memory.** *Nat Rev Neurosci* 2010, **11:**675-681.

14. Kallarackal AJ, Kvarta MD, Cammarata E, Jaberi L, Cai X, Bailey AM, Thompson SM: **Chronic stress induces a selective decrease in AMPA receptor-mediated synaptic excitation at hippocampal temporoammonic-CA1 synapses.** *J Neurosci* 2013, **33:**15669-15674.

15. Pittenger C, Duman RS: **Stress, depression, and neuroplasticity: a convergence of mechanisms.** *Neuropsychopharmacology* 2008, **33:**88-109.

16. Weiss C, Sametsky E, Sasse A, Spiess J, Disterhoft JF: **Acute stress facilitates trace eyeblink conditioning in C57BL/6 male mice and increases the excitability of their CA1 pyramidal neurons.** *Learn Mem* 2005, **12:**138-143.

17. Suenaga T, Morinobu S, Kawano K, Sawada T, Yamawaki S: **Influence of immobilization stress on the levels of CaMKII and phospho-CaMKII in the rat hippocampus.** *Int J Neuropsychopharmacol* 2004, **7:**299-309.

18. Whitehead G, Jo J, Hogg EL, Piers T, Kim DH, Seaton G, Seok H, Bru-Mercier G, Son

GH, Regan P, et al: **Acute stress causes rapid synaptic insertion of Ca2+ -permeable AMPA receptors to facilitate long-term potentiation in the hippocampus.** *Brain* 2013, **136:**3753-3765.

19. Kim JW, Ko MJ, Gonzales EL, Kang RJ, Kim DG, Kim Y, Seung H, Oh HA, Eun PH, Shin CY: **Social support rescues acute stress-induced cognitive impairments by modulating ERK1/2 phosphorylation in adolescent mice.** *Sci Rep* 2018, **8:**12003.

20. El Gaamouch F, Buisson A, Moustie O, Lemieux M, Labrecque S, Bontempi B, De Koninck P, Nicole O: **Interaction between alphaCaMKII and GluN2B controls ERK-dependent plasticity.** *J Neurosci* 2012, **32:**10767-10779.

21. Paul S, Nairn AC, Wang P, Lombroso PJ: **NMDA-mediated activation of the tyrosine phosphatase STEP regulates the duration of ERK signaling.** *Nat Neurosci* 2003, **6:**34-42.

22. Bluthgen N, van Bentum M, Merz B, Kuhl D, Hermey G: **Profiling the MAPK/ERK dependent and independent activity regulated transcriptional programs in the murine hippocampus in vivo.** *Sci Rep* 2017, **7:**45101.

23. Yoon SH, Chung G, Song WS, Oh SP, Kim J, Kim SJ, Kim M-H: **Altered GABAergic inhibition in CA1 pyramidal neurons modifies despair-like behavior in mice.** *BioRxiv* 2021**:** https://doi.org/10.1101/2020.02.18.953786.

24. Pullan LM, Powel RJ: **Comparison of binding at strychnine-sensitive (inhibitory glycine receptor) and strychnine-insensitive (N-methyl-D-aspartate receptor) glycine binding sites.** *Neurosci Lett* 1992, **148:**199-201.

25. Jakubik J, Bacakova L, El-Fakahany EE, Tucek S: **Positive cooperativity of acetylcholine and other agonists with allosteric ligands on muscarinic acetylcholine receptors.** *Mol Pharmacol* 1997, **52:**172-179.
